# Relevance of ineffective esophageal motility to striated esophageal muscle contraction: Studies with high‐resolution manometry

**DOI:** 10.1002/kjm2.12884

**Published:** 2024-08-01

**Authors:** Jui‐Sheng Hung, Wei‐Yi Lei, Ming‐Wun Wong, Chih‐Hsun Yi, Tso‐Tsai Liu, Chien‐Lin Chen

**Affiliations:** ^1^ Department of Medicine Hualien Tzu Chi Hospital, Buddhist Tzu Chi Medical Foundation and Tzu Chi University Hualien Taiwan; ^2^ Institute of Medical Sciences Tzu Chi University Hualien Taiwan

**Keywords:** esophageal motility, high‐resolution manometry, ineffective esophageal motility, striated muscle contraction

## Abstract

Striated esophageal muscle contraction (SEC) is important for pharyngeal swallowing and deglutition augmentation against aspiration. Its clinical relevance is unclear in patients with ineffective esophageal motility (IEM). In this study, we aimed to characterize and compare SEC in consecutive patients with and without IEM. All eligible patients were evaluated for SEC, primary and secondary peristalsis using high‐resolution manometry (HRM) with one mid‐esophageal injection port. Primary peristalsis was assessed with 10 5‐mL liquid swallows and multiple rapid swallows (MRS), while secondary peristalsis was performed with rapid air injections of 20 mL. All peristatic parameters of HRM were measured, and SEC and its contractile integral (SECI) were evaluated. One hundred and forty patients (59.3% women, mean age 46.1 ± 13.1 years) were included. There was no difference in SECI between patients with and without IEM (*p* = 0.91). SECI was also similar between patients with and without secondary peristalsis for IEM (*p* = 0.63) or normal motility (*p* = 0.80). No difference in SECI was seen between patients with and without MRS for IEM (*p* = 0.55) or normal motility (*p* = 0.88). SECI was significantly higher in male patients than female patients in IEM patients (*p* = 0.01). SECI significantly correlated with age in patients with normal motility (*r* = −0.31, *p* = 0.01). Aging may have a negative impact on SEC in patients with normal motility, while gender difference in SECI occurs in IEM patients. Neither secondary peristalsis nor MRS influences SECI.

## 1 INTRODUCTION

Primary peristalsis transports esophageal bolus after swallowing, whereas distension‐induced or secondary peristalsis functions to maintain an empty esophagus by clearing refluxate from the stomach^1^ or residual food bolus after swallowing. Esophageal peristalsis in both striated muscle and distal smooth muscle parts is controlled by brainstem nuclei. Striated muscle is directly innervated by vagal efferents from the nucleus ambiguous, whereas smooth muscle is modulated by myenteric gangions and motor neurons with connection to the dorsal motor nucleus of the vagus nerve.^2^ The role of striated esophageal muscle contractions (SEC) is not only responsible for proximal swallowing but also for preventing the risk aspiration against the entry of acid reflux into the pharynx. Impaired SEC have been demonstrated in elderly and achalasia.^3^ Physiological implications of SEC are also related to bolus volume and consistency, biomechanics of pharyngeal swallowing, and pharyngo‐esophageal fluid dynamics.

The esophagus has two separate contraction patterns, one above and one below the proximal transition zone, properly coordinated during normal bolus transport.^4^ Although there is a possibility of independent contractions between proximal and distal esophagus, we hypothesized that the presence of ineffective esophageal motility (IEM) may impact SEC. Therefore, the aim of this study was to evaluate and compare SEC in consecutive patients with and without IEM, and also correlated SEC to primary and secondary peristalsis.

## 2 METHODS

### 2.1 Subjects

This prospective study included patients with normal motility and those with ineffective esophageal motility after high‐resolution manometry (HRM) studies. All tracings were corrected from 140 consecutive patients (59.3% women, mean age 46.1 ± 13.1 years) referred for HRM. The diagnosis of IEM was made according to Chicago 4.0.^5^ Esophageal symptoms were recorded in each patient. The exclusion criteria in this study include the following conditions: (1) esophageal strictures, (2) previous gastrointestinal surgery, (3) presence of systemic diseases that might interfere with esophageal motility, (4) chronic use of medications known to affect esophageal motility, and (5) significant esophageal motility disorders (achalasia, hypercontractile disorders, esophagogastric junction outlet obstruction). Demographic characteristics and esophageal symptoms of all patients were shown in Table 1.

**TABLE 1:** Demographic and clinical characteristics of all patients.

Characteristics	Patients (*n* = 140)
Age (years)	46.1 (1.1)
Gender (Female, %)	59.3
Symptoms (%)	
Heartburn	12.1
Chest pain	5
Acid regurgitation	54.3
Cough	23.6
Dysphagia	11
Globus	8.6
Hiccup	5

All subjects agreeing to participate signed an informed consent form of this study, which was approved by the Research Ethics Committee of Hualien Tzu Chi Hospital, Buddhist Tzu Chi Medical Foundation, Hualien, Taiwan.

### 2.2 HRM protocol

After an overnight fast, a catheter with 22‐channel pressure sensors containing with one air injection port in the mid‐esophagus (MMS, HRM, Enschede, The Netherlands) was inserted through an anesthetized nostril. Nasal passage of the HRM assembly was passed into the esophagogastric portion, and was then placed with at least three distal sensors positioned in the stomach to record the data from hypopharynx to the stomach. The luminal diameter for each perfusion capillary was 0.4 mm, while the total diameter was 4.7 mm by perfusing with ambient distilled water at a rate of 0.15 mL/min. All data were recorded using external pressure transducers (Argon Medical Devices, Plano, TX). Swallowing event was detected using most proximal channel of the catheter, which assisted the differentiation of primary and secondary peristalsis. A total of 10 saline (5 mL) swallows at 30‐s intervals were performed in each patient in a semi‐recumbent position. Then, three multiple rapid swallows (MRS) (one swallow every 2–3 s) were done with 10 mL of water injected steadily into their mouths through a syringe.^6^ Each MRS consists at least four 2‐mL water swallows in a rapid fashion, with ≤4‐s interval between swallows.

Rapid air distension was done with 10 times of 20 mL bolus mid‐esophageal air injections in all participants. The interval of each stimulus was at least 30 s. After finishing each trial of rapid air injection, all patients were asked to have a dry swallow for clearing any remaining air in order to reduce swallow attempt through entire esophageal stimulation. The effectiveness of secondary peristaltic response (%) and parameters of HRM were recorded.

### 2.3 Data analysis

HRM data were analyzed using comprehensive software (MMS Database software, MMS, Enschede, The Netherlands). Primary and secondary peristalsis were judged as successful if the typical peristaltic pattern of pressure topography was demonstrated on HRM. Peristaltic frequency (%) of either primary or secondary peristalsis was therefore calculated as the number of successful responses divided by total primary or secondary peristalsis (10 times, each). The distal contractile integral (DCI) represented contractile vigor of esophageal peristalsis, which was calculated by data analysis software (MMS). Analysis of MRS included completeness of esophageal body inhibition and lower esophageal sphincter (LES) relaxation, whereas DCI after MRS was measured immediately after the last MRS swallow.^7^ Normal MRS response with augmentation was judged.^8^ Whereas SEC and its contractile integral (SECI) were evaluated and recorded in the esophageal contractile isocontour region between the distal upper esophageal sphincter margin and the nadir of the esophageal transition zone.

### 2.4 Statistical analysis

All HRM data between patients with normal motility and IEM were compared with a paired test and expressed as mean ± standard error of the mean (SEM). Evaluation of data normality was performed using D'Agostino's *K*‐squared test. Tests for the proportionality between groups were used Chi‐square analysis and Fisher's exact tests. Data for correlation were evaluated by the Pearson correlation coefficient. A *p‐*value <0.05 was considered significant. Statistical analyses were performed with SPSS 22.0 for Windows (SPSS, Chicago, IL).

## 3 RESULTS

### 3.1 Impact of esophageal peristalsis on the integrity of striated muscle contraction

One hundred and forty patients (59.3% women, mean age 46.1 ± 13.1 years) were included, of whom there were 70 patients with IEM and 70 patients with normal motility. There was no difference for age or gender between two groups of the patients. SECI was comparable between patients with and without IEM (*p* = 0.91) (Table 2). There was no difference in SECI between patients with and without secondary peristalsis for IEM (*p* = 0.71) or normal motility (*p* = 0.88). Moreover, SECI was similar between patients with and without MRS augmentation for patients with IEM (*p* = 0.54) or normal motility (*p* = 0.87) (Table 3).

**TABLE 2:** Age, gender, SECI between patients with IEM and normal motility.

	IEM	Normal motility	*p*‐Value
Age (years)	45.9 (1.6)	46.3 (1.7)	0.87
Gender (female, %)	60	59	0.9
SECI (mmHg.s.cm)	368.2 (37.3)	373.7 (32.9)	0.91

Abbreviations: IEM, ineffective esophageal motility; SECI, striated esophageal contractile integral; Data are expressed as mean (SEM).

**TABLE 3:** Relevance of gender, MRS, and secondary peristalsis to SECI.

SECI		IEM	*p*‐Value	Normal motility	*p*‐Value
Gender	Male	503.4 (78.9)	0.01	377.2 (47.7)	0.93
	Female	278.2 (26.1)		371.1 (45.3)	
MRS	Present	354.5 (45.1)	0.54	378.5 (57.8)	0.87
	Absent	408.0 (65.1)		367.4 (34.6)	
Secondary peristalsis	Present	353.1 (57.1)	0.71	371.1 (44.7)	0.88
	Absent	381.8 (49.6)		379.9 (35.4)	

*Note*: Data are expressed as data (SEM).

Abbreviations: IEM, ineffective esophageal motility; MRS, multiple rapid swallows; SECI, striated esophageal contractile integral.

### 3.2 Association of clinical features to the integrity of striated muscle contraction

**FIGURE 1:**
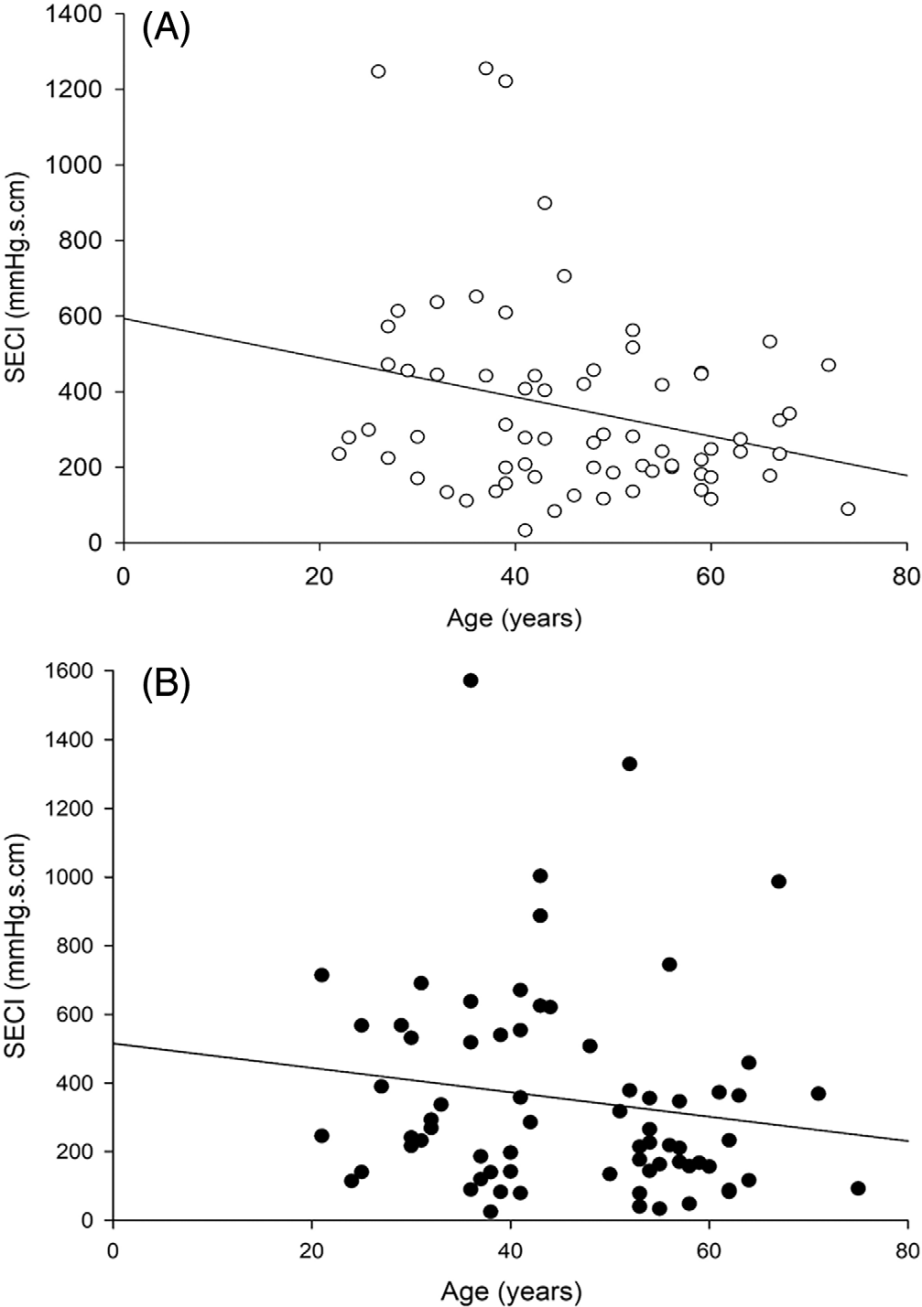
There is a significantly negative correlation between SECI and age in patients with normal motility (*r* = −0.31, *p* = 0.01) (A); there is no correlation between SECI and age in patients with IEM (*r* = −0.14, *p* = 0.24) (B). Each dot represents individual value for each patient. IEM, ineffective esophageal motility; SECI, striated esophageal contractile integral.

SECI was significantly higher in male patients than female patients in IEM patients (*p* = 0.01), but not in patients with normal motility group (*p* = 0.93) (Table 3). SECI significantly correlated with age in patients with normal motility (*r* = −0.31, *p* = 0.01) (Figure 1A), but not seen in patients with IEM (*r* = −0.14, *p* = 0.24) (Figure 1B).

## 4 DISCUSSION

In this study, we report that both patients with and without IEM have similar contraction vigor for esophageal striated muscle. There was no difference in SECI between patients with and without secondary peristalsis in both groups of the patients. Similarly, contraction vigor of esophageal striated muscle was also comparable between patients with and without MRS in both groups of the patients. Our data demonstrated that male IEM patients had greater SECI than female IEM patients, but no gender difference was observed for those with normal motility. Furthermore, there was a significantly negative correlation between SECI and age only for patients with normal motility.

The control of SEC is entirely from the central nervous system, by which every swallowed bolus inside the esophagus triggers the onset of the esophageal phase of swallowing.^9^ The propagation of the SEC from proximal esophagus progressively slows down and become weaker until ending at the proximal border of the esophageal transition zone. Consequently, it is followed by another contraction wave arising distally via myenteric plexus in that segment, which functions for transporting swallowed bolus into the smooth‐muscle part of lower esophagus.^4^ These two contractions are properly coordinated for normal bolus transport.^4^ Esophageal bolus swallowing activates vagal sensory neurons and evokes peristalsis via vagal dorsal motor neurons connected to the smooth muscle. In a previous study, spontaneous distal hypotensive esophageal motility as induced by sildenafil was associated with an increase in proximal esophageal motility.^10^ However, other studies did not reveal any alteration of proximal esophageal contractions after pharmacological induction of ineffective distal contractions.^11^ In our study, we didn't find any difference in contraction vigor of esophageal striated muscle between patients with and without IEM. It appears that acute modulation in response to induce distal esophageal hypomotility may potentially results in proximal or striated portion esophageal hypercontraction owing to compensation for promoting esophageal bolus clearance. However, since IEM may indicate chronic status of esophageal hypomotility, it is plausible that striated portion esophagus may be adjusted into the contractility at least comparable to that of patients with normal motility, which was shown in our study.

Secondary peristalsis can be successfully triggered by different intraesophageal stimuli.^12^, ^13^ The intrinsic reflex that modulates secondary peristalsis includes a vagal afferent pathway in which both mucosal and intramuscular mechanoreceptors contribute to generate this peristalsis.^14^, ^15^ Additionally, esophageal peristalsis can be also mediated by local neuromuscular reflex inside enteric nervous system and vagovagal reflex.^16^ Therefore, lack of any difference in SECI between patients with and without secondary peristalsis may suggest the evidence that activation of SEC directly from central mechanism which is different from that of secondary peristalsis requiring complex interactions including central and peripheral neuromuscular coordination.

MRS is a commonly provocative maneuver for HRM.^7^ MRS induces a profound inhibition of the esophageal body and LES tone during sequential swallows. After the final swallow of the MRS sequence, robust esophageal body contraction with regained LES tone are normally present. Therefore, intact neural connections and smooth muscle function are required for an intact MRS response.^7^ In this study, we didn't observe any difference in SECI between patients with and without MRS in either group, suggesting that modulation of SEC is different from that of MRS which is relevant to sensorimotor function of esophageal smooth muscle.

It is interesting to demonstrate in patients with normal motility that reduced SECI is associated with aging. It has been recently reported that there is a positive correlation between age and DCI of esophageal smooth muscle^17^; however, other studies have conflicted results regarding DCI in elderly patients.^18^ A recent study has demonstrated that aging has negative impact on deglutitive SECI in elderly adults.^19^ Our study has also shown lower SECI in aging patients, but its clinical implication awaits to be established. The explanation may be due to muscle weakness or altered sensorimotor function owing to aging process. It is important to know that the function of SEC may protect against aspiration, increasing awareness of the changes in such motility alteration could potentially help establish the strategy by monitoring pharyngeal augmentation in order to prevent aspiration in the elderly population.

In this study, it was observed in IEM patients that SECI was higher in male patients than female patients, but no gender difference was found for patients with normal motility. In a previous study on esophageal motility of healthy volunteers, it revealed that there was no difference in proximal and distal contractile integral between men and women.^20^ The discrepancy may be caused by different studied subjects, sample size, and different methodology; however, current notion and its clinical relevance await to be elucidated in patients with other motility disorders.

There are some limitations in this study that need to be addressed. First, we didn't perform and evaluate bolus clearance characteristics with SEC to explore more comprehensive peristaltic physiology relevant to the contraction of striated esophagus. Second, this study explored patients with and without IEM; however, it remains to be determined whether current results can be observed in healthy adults and patients with other motility disorders. Finally, this study was performed at a tertiary referral center, which could limit the generalizability of current findings to general population or those using other HRM equipment.

In summary, the presence of IEM per se has no impact on the contractility of striated esophagus. Aging may have a negative influence on the contractile vigor of striated esophagus in patients with normal motility. In patients with IEM, higher contractile vigor can be found in men than woman. It confirms the notion that neither secondary peristalsis nor MRS correlates to the contractility of striated esophageal muscle contraction. Assessment of striated esophageal contractility may have certain clinical utility and implication when doing routine HRM for esophageal motility, particularly in patients with old age and disordered swallowing.

## CONFLICT OF INTEREST STATEMENT

All authors declare no conflict of interest.

## References

[1] Helm JF , Dodds WJ , Pelc LR , Palmer DW , Hogan WJ , Teeter BC . Effect of esophageal emptying and saliva on clearance of acid from the esophagus. N Engl J Med. 1984;310(5):284–288. 6690951 10.1056/NEJM198402023100503

[2] Clouse RE , Diamant NE . Esophageal motor and sensory function and motor disorders of the esophagus. In: Feldman M , Friedman LS , Sleisenger MH , editors. Gastrointestinal and liver Disease: pathophysiology, diagnosis, management. Volume 1. 7th ed. Philadelphia: WB Saunders; 2002. p. 561–598.

[3] Edeani FO , Kern M , Sanvanson P , Mei L , Yu ES , Shaker R . Sa1213: further characterization of striated muscle esophagus contractile function in health and disease. Gastroenterology. 2022;162:S‐349–S‐350.

[4] Li M , Brasseur JG , Dodds WJ . Analyses of normal and abnormal esophageal transport using computer simulations. Am J Phys. 1994;266(4 Pt 1):G525–G543. 10.1152/ajpgi.1994.266.4.G525 8178991

[5] Yadlapati R , Pandolfino JE , Fox MR , Bredenoord AJ , Kahrilas PJ . What is new in Chicago classification version 4.0? Neurogastroenterol Motil. 2021;33(1):e14053. 33340190 10.1111/nmo.14053 PMC8098672

[6] Savojardo D , Mangano M , Cantù P , Penagini R . Multiple rapid swallowing in idiopathic achalasia: evidence for patients' heterogeneity. Neurogastroenterol Motil. 2007;19(4):263–269. 17391242 10.1111/j.1365-2982.2006.00886.x

[7] Fornari F , Bravi I , Penagini R , Tack J , Sifrim D . Multiple rapid swallowing: a complementary test during standard oesophageal manometry. Neurogastroenterol Motil. 2009;21(7):718‐e41. 19222762 10.1111/j.1365-2982.2009.01273.x

[8] Shaker A , Stoikes N , Drapekin J , Kushnir V , Brunt LM , Gyawali CP . Multiple rapid swallow responses during esophageal high‐resolution manometry reflect esophageal body peristaltic reserve. Am J Gastroenterol. 2013;108(11):1706–1712. 24019081 10.1038/ajg.2013.289 PMC4091619

[9] Lang IM . Brain stem control of the phases of swallowing. Dysphagia. 2009;24(3):333–348. 19399555 10.1007/s00455-009-9211-6

[10] Costa TV , Dantas RO . Proximal esophageal contraction after induction of ineffective distal contraction by sildenafil in healthy volunteers. Ann Gastroenterol. 2020;33(1):19–24. 31892793 10.20524/aog.2019.0443 PMC6928477

[11] Kim HS , Conklin JL , Park H . The effect of sildenafil on segmental oesophageal motility and gastro‐oesophageal reflux. Aliment Pharmacol Ther. 2006;24(7):1029–1036. 16984496 10.1111/j.1365-2036.2006.03091.x

[12] Schoeman MN , Holloway RH . Stimulation and characteristics of secondary oesophageal peristalsis in normal subjects. Gut. 1994;35(2):152–158. 8307463 10.1136/gut.35.2.152 PMC1374487

[13] Chen CL , Szczesniak MM , Cook IJ . Oesophageal bolus transit and clearance by secondary peristalsis in normal individuals. Eur J Gastroenterol Hepatol. 2008;20(12):1129–1135. 18989139 10.1097/MEG.0b013e328303bff1

[14] Lang IM , Medda BK , Shaker R . Mechanisms of reflexes induced by esophageal distension. Am J Physiol Gastrointest Liver Physiol. 2001;281(5):G1246–G1263. 11668034 10.1152/ajpgi.2001.281.5.G1246

[15] Kravitz JJ , Snape WJ Jr , Cohen S . Effect of thoracic vagotomy and vagal stimulation on esophageal function. Am J Phys. 1978;234(4):E359–E364. 10.1152/ajpendo.1978.234.4.E359 645850

[16] Park H , Conklin JL . Neuromuscular control of esophageal peristalsis. Curr Gastroenterol Rep. 1999;1(3):186–197. 10980948 10.1007/s11894-999-0033-3

[17] Djinbachian R , Marchand E , Yan W , Bouin M . Effects of age on esophageal motility: a high‐resolution manometry study. J Clin Med Res. 2021;13(8):413–419. 34527096 10.14740/jocmr4576 PMC8425793

[18] Shim YK , Kim N , Park YH , Lee JC , Sung J , Choi YJ , et al. Effects of age on esophageal motility: use of high‐resolution esophageal impedance manometry. J Neurogastroenterol Motil. 2017;23(2):229–236. 28163259 10.5056/jnm16104 PMC5383117

[19] Kern M , Edeani FO , Sanvanson P , Mei L , Shaker R . Su1244: the effect of aging on the interaction between striated esophageal flow dynamics and contractile vigor. Gastroenterology. 2022;162:S‐557–S‐558.

[20] Costa TV , Dantas RO . Esophageal motility in men and women evaluated by high‐resolution manometry. Arq Gastroenterol. 2017;54(2):145–147. 28273277 10.1590/S0004-2803.201700000-10

